# Non-pharmacological interventions for delirium in the pediatric population: a systematic review with narrative synthesis

**DOI:** 10.1186/s12887-024-04595-4

**Published:** 2024-02-12

**Authors:** Kyua Kim, Ju Hee Jeong, Eun Kyoung Choi

**Affiliations:** 1 Department of Nursing, Yonsei University Graduate School & Pediatric Intensive Care Unit, Severance Hospital, 50-1 Yonsei-Ro, Seodaemun-Gu, Seoul, 03722 South Korea; 2 Department of Nursing, Yonsei University Graduate School & Emergency Nursing, Severance Hospital, 50-1 Yonsei-Ro, Seodaemun-Gu, Seoul, 03722 South Korea; 3 College of Nursing &, Mo-Im Kim Nursing Research Institute, Yonsei University, 50-1 Yonsei-Ro, Seodaemun-Gu, Seoul, 03722 South Korea

**Keywords:** Critical care, Delirium, Pediatric, Intensive care unit, Systematic review

## Abstract

**Background:**

Delirium is a serious complication experienced by hospitalized children. Therefore, preventive management strategies are recommended for these patients. However, comprehensive analyses of delirium interventions in children remain insufficient. Specifically, this systematic review aimed to summarize non-pharmacological interventions for pediatric delirium, addressing the urgent need for a comprehensive understanding of effective strategies. We also explored frequently measured outcome variables to contribute evidence for future research on delirium outcomes in children.

**Methods:**

This systematic review searched articles from PubMed, Web of Science, Cumulative Index to Nursing and Allied Health Literature, and Excerpta Medica databases. The eligibility criteria were formed under the population, intervention, comparator, outcome, and study design framework. Studies were included if they involved (1) children aged under 18 years receiving hospital care, (2) non-pharmacological delirium interventions, (3) comparators involving no intervention or pharmacological delirium interventions, and (4) outcomes measuring the effectiveness of non-pharmacological delirium interventions. Only peer-reviewed articles published in English were included.

**Results:**

Overall, 16 studies were analyzed; of them, 9 assessed non-pharmacological interventions for emergence delirium and 7 assessed interventions for pediatric delirium. The intervention types were grouped as follows: educational (*n* = 5), multicomponent (*n* = 6), and technology-assisted (*n* = 5). Along with pediatric and emergence delirium, the most frequently measured outcome variables were pain, patient anxiety, parental anxiety, pediatric intensive care unit length of stay, agitation, analgesic consumption, and postoperative maladaptive behavior.

**Conclusions:**

Non-pharmacological interventions for children are effective treatments without associated complications. However, determining the most effective non-pharmacological delirium intervention for hospitalized children based on current data remains challenging.

**Supplementary Information:**

The online version contains supplementary material available at 10.1186/s12887-024-04595-4.

## Background

Delirium is a neuropsychiatric disorder that disrupts cerebral function due to underlying diseases or critical care treatment [1]. It manifests as acute cognitive impairment, encompassing changes in attention and awareness, sleep cycle disturbances, agitation, and hallucinations, occurring in 20–70% of hospitalized patients of all ages [2, 3]. Infants and preschool-aged children exhibit delirium symptoms similar to adults [4].

The epidemiology of delirium in hospitalized patients varies across clinical scenarios, with common occurrences in medical-surgical wards, intensive care units (ICUs), postoperative populations, and emergency departments. The prevalence ranges from 2.1 to 94.8% in adults [5]. Delirium also occurs in hospitalized children in diverse settings, with pediatric delirium estimated to occur in 34% of critical care admissions [6], and emergence delirium in over 40% of patients in postoperative surgery care [7].

Pediatric delirium, primarily observed in the pediatric ICU (PICU), manifests in subtypes, including hypoactive, hyperactive, and mixed delirium, with the mixed type being the most prevalent [1]. However, current delirium assessment tools commonly encounter challenges in accurately distinguishing between these subtypes, resulting in unrecognized and undiagnosed cases and frequent omission of active screening in clinical settings [8]. Risk factors for pediatric delirium include age under 2 years, mechanical ventilator use for 48 h or longer, immobilization, impaired baseline cognitive function, metabolic dysfunction, hypoxia, benzodiazepine use, and restraint use in the PICU [9–11].

Furthermore, children are at a risk of developing emergence delirium after surgical intervention. If children exhibit several cognitive and behavioral dysregulations, such as non-purposeful resistive movements, kicking, pulling, flailing, lack of eye contact, and awareness of surroundings [12, 13], within 45 min of surgery anesthesia, they meet delirium criteria upon transfer from the post-anesthesia care unit (PACU) to the general ward or ICU [14]. Risk factors for emergence delirium span across three categories: patient-related, anesthesia-related, and surgical factors [12]. In summary, children encounter various risk factors for delirium across all phases of hospital care.

Regardless of the timing and circumstances of a child’s delirium experience, pediatric and emergence delirium experience in a hospital causes significant short- and long-term health outcomes. Children with pediatric delirium can experience prolonged mechanical ventilation and length of hospital stay, leading to excess mortality [15]. It increases PICU costs by up to 85% [16]. Additionally, pediatric delirium is associated with a decline in post-discharge health-related quality of life for children under 5 years [17]. Traube [18] suggested long-term research and follow-up studies in PICU survivors with pediatric delirium to investigate the correlation between pediatric delirium and post-intensive care syndrome. Moreover, children with emergence delirium develop more severe behavioral changes 1 week after surgery [19]. Given these critical problems, healthcare professionals must actively develop an integrative and holistic intervention for all hospitalized children at risk of pediatric and emergence delirium.

To date, healthcare professionals have explored pharmacological and non-pharmacological approaches for pediatric and emergence delirium [1, 2, 11, 13]. Typical or atypical antipsychotics are used as pharmacological interventions for delirium, even though their use for delirium treatment is not approved by the United States Food and Drug Administration, and these drugs are not licensed for use in children. Antipsychotics, including haloperidol, olanzapine, risperidone, and quetiapine, are used as first-line pharmacological treatments [20]. Dexmedetomidine, magnesium sulfate, and melatonin have also been used in adults and children with delirium [21–23]. Pharmacological interventions in children yield some positive outcomes; however, the associated side effects cannot be overlooked. Children may experience tachycardia, hypotension, sedation, low-threshold seizures, and neuroleptic malignant syndrome with the use of antipsychotic drugs [20]. The side effects of haloperidol outweigh its therapeutic effects even at low plasma concentrations [24]. Moreover, the effect of melatonin use on children is still not fully understood [25]. Recent research reports that antipsychotics are less effective than non-pharmacological interventions in critically ill children [26].

Multicomponent non-pharmacological interventions have demonstrated positive outcomes in reducing the duration and occurrence of delirium in adult ICU and general ward settings [27, 28].

Similarly, non-pharmacological interventions have been attempted in the pediatric population.

These include educational interventions for parents, children, and healthcare professionals [29–31], playing music and mothers’ voices for children [32–34], and providing weighted blankets as an intervention [35]. Furthermore, multicomponent interventions have been explored in children [36].

Given the need for a comprehensive analysis of delirium interventions in children, we aimed to summarize the effectiveness and limitations of non-pharmacological interventions for delirium in children in this systematic review. Moreover, we aimed to identify frequently measured outcome variables in non-pharmacological delirium intervention research. Herein, we present a narrative synthesis to build evidence for future delirium research in children.

## Materials and methods

### Search method

An electronic database search was performed using PubMed, Web of Science, Cumulative Index to Nursing and Allied Health Literature (CINAHL), and Excerpta Medica database (EMBASE) on May 22, 2021. The search employed three-part keywords: population, diseases, and intervention. Keywords such as “Child,” “Children,” “Pediatric,” “Newborn,” “Infant,” “Delirium,” and “Intervention” were combined. Medical Subject Heading terms from each database were also included in the literature search. A librarian (NWK) with expertise in systematic review search processes reviewed the search keywords. Further, no specific time constraints were applied to the publication date of the articles selected, allowing for a comprehensive and inclusive analysis of relevant literature on non-pharmacological interventions for delirium in children. All the literature searched from the four databases was uploaded to EndNote (Clarivate, London, UK), a web-based reference management software, and duplicates were removed.

### Eligibility criteria

The population, intervention, comparator, outcome, and study design framework guided eligibility criteria. Studies were included if they involved the following: (1) children aged < 18 years receiving hospital care, (2) non-pharmacological delirium interventions, (3) comparators involving no intervention or pharmacological delirium interventions, and (4) outcomes measuring the effectiveness of non-pharmacological delirium interventions. Only peer-reviewed articles published in English were included. The study designs included randomized controlled trials (RCTs), cohort studies, and quality improvement projects. Owing to the early stage of pediatric delirium research, quality improvement projects were included in the analysis.

Exclusion criteria involved studies: (1) that did not address the phenomenon of interest, (2) in which interventions were conducted in adults, (3) that were written in a language other than English, (4) that were review articles, (5) that were case studies and protocols, (6) with unavailable full text, and (7) that were duplicates. Two reviewers (KK and JHJ) independently evaluated each article using the eligibility criteria. When opinions differed regarding the final selection of the study, a third author (EKC) intervened to facilitate the discussion.

### Quality appraisal

Two authors independently performed quality appraisals using the Preferred Reporting Items for Systematic Reviews and Meta-Analyses checklist. Two authors independently conducted a risk-of-bias assessment for included articles using the Joanna Briggs Institute Critical Appraisal Checklist for RCTs and Cohort Studies, the Cochrane Risk of Bias, and the Quality Improvement Minimum Quality Criteria Set [37–39].

### Data extraction

**Fig. 1:**
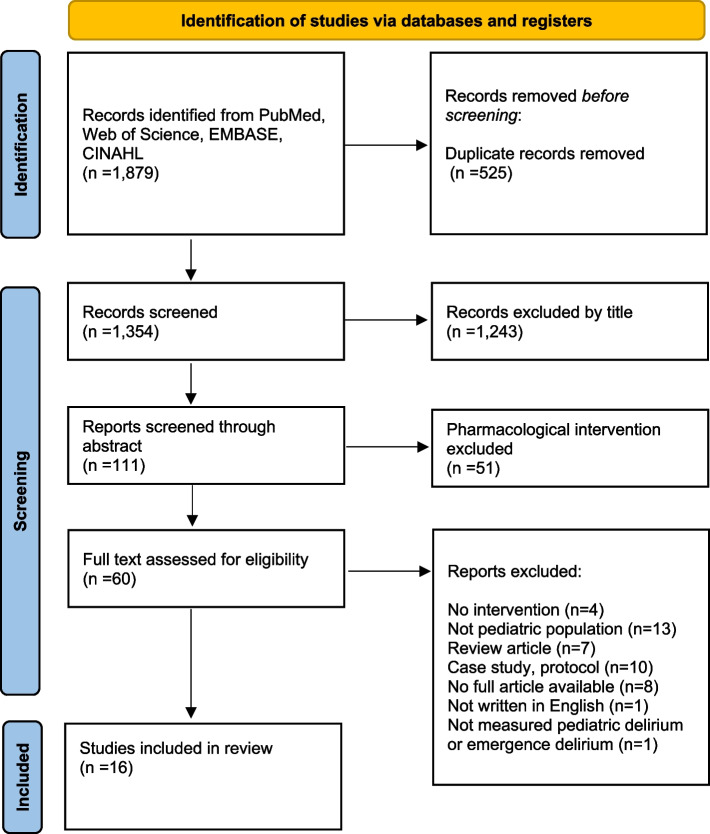
PRISMA flow diagram

Two authors (KK and JHJ) collected data from 1,879 records across PubMed, Web of Science, EMBASE, and CINAHL. Titles and abstracts were independently screened based on shared inclusion and exclusion criteria. When opinions differed during the final study selection, reviewers engaged in further discussion to achieve consensus. When consensus was unattainable, a third author (EKC) intervened to facilitate resolution. Ultimately, this systematic review included 16 studies (Fig. 1) [40].

### Synthesis

Owing to heterogeneity in non-pharmacological interventions, age-related differences in the pediatric population, and variations in outcome variables and measurement instruments across studies, a meta-synthesis was not feasible in this systematic review. A narrative synthesis of the systematic review was performed in the final analysis of the included studies to address this limitation. Guidance on conducting narrative synthesis in systematic reviews was consulted, and techniques such as grouping and clustering, thematic analysis, critical reflection, exploration of relationships within and between studies, idea webbing, and conceptual mapping were employed to comprehensively overview the included studies [41]. Consequently, the narrative synthesis of this systematic review suggests indications for future delirium in children.

### Objective

This systematic review aimed to summarize non-pharmacological interventions for delirium in children to outline current research trends and future directions. This review also explored frequently measured outcome variables in this field of research.

## Results

### Study characteristics

Table 1 presents the characteristics and summary of the studies included in this systematic review. Non-pharmacological interventions were categorized into two groups: interventions for emergence delirium (nine studies; 56.3%) or pediatric delirium (seven studies; 43.7%). Eight RCTs and one cohort study examined interventions for emergence delirium. Pediatric delirium interventions were explored in two RCTs, one cohort study, and four quality improvement projects. Only 1 study [42] was published before 2010, with the remaining 15 studies published between 2015 and 2021.

**Table 1:** Summary of studies included in the systematic review

Type	Author (year)	Design	Sample size	Age (year)	Intervention	Primary outcome	Secondary outcome	Result
PD	Cloedt et al. (2022) [43]	Cohort study	314	Under 21	PAD bundle	Rate of delirium detection, pain, PD, level of agitation, cumulative doses of analgesics and sedatives per day	N/A	Detection of PD increased from 4% to 31.9% *(p* < 0.001)
PD	Garcia Guerra et al. (2021) [33]	RCT	60	1M-16	Music intervention	Patient’s sedation status	PD	No change in PD
PD	Rohlik et al. (2021) [44]	QI	780	N/A	BED bundle education, feedback, and implementation	Delirium assessment documentation rate, impact of delirium education, perceived confidence of delirium management, perceived barrier of delirium management	N/A	Increased delirium assessment documentation, nursing confidence in delirium management, and lowered perceived assessment barriers (*p* < 0.05)
PD	Silver and Traube (2019) [45]	QI	15	2–12	Delirium prevention toolkit and PICU journal	Family satisfaction	N/A	Increased satisfaction of parents and family
PD	Kawai et al. (2019) [46]	QI	8	1–10	BED bundle and noise pollution reduction	Hourly dB readings, the impact of pediatric delirium bundle	N/A	Lowered noise pollution in PICU (*p* < 0.01)
ED	Byun et al. (2018) [32]	RCT	66	2–8	Mother’s voice	Emergence delirium	Postoperative pain	PAED score decreased (*p* = 0.006)
ED	Nakamura et al. (2018) [47]	RCT	100	18M-8	Unilateral right-side stimulation of HT7 acupuncture point	Emergence delirium	Incidence and severity of emergence delirium, PACU length of stay, postoperative pain	No change in EA incidence
PD	Rohlik et al. (2018) [30]	RCT	2073	5–17	Delirium education	PD, delirium assessment documentation rate, barriers to delirium assessment	N/A	Increased PD assessment and documentation rate
ED	Zhong et al. (2018) [31]	RCT	69	3–6	Pre-operative visit	EA	Parental separation scale, mask acceptance scale, Aldrete score (time to discharge)	Lowered EA and propofol administration (*p* < 0.05)
PD	Simone et al. (2017) [48]	QI	1875	0–24	ICU bundle implementation	Screening compliance, delirium prevalence, staff delirium knowledge and attitudes	N/A	Effective for improving delirium screening, detection, and treatment
ED	Song et al. (2017) [34]	RCT	127	2–8	Mother’s voice	EA	Awakening time, PACU length of stay	Lowered EA, awakening time, and PACU length of stay
ED	Ohashi et al. (2016) [49]	RCT	40	1–6	The ultrasound-guided II/IH nerve block	ED	Postoperative pain	Not effective in lowering emergence delirium. Effective in lowered use of intra-operative sevoflurane
ED	Bailey et al. (2015) [29]	RCT	93	2–10	Video-based PPIA preparation	Patient anxiety	Parental anxiety, child observational measures, emergence delirium, postoperative pain	No change in emergence delirium However, higher self-efficacy was observed in helping their children in the OR. (*p* = 0.03, odds ratio [95% confidence interval] = 1.69 [1.07–2.87])
ED	Hilly et al. (2015) [50]	Cohort study	56	3–18	Pre-operative preparation workshop	Patient anxiety, parental anxiety, postoperative maladaptive behavior	PACU morphine consumption, PACU length of stay, emergence delirium	Patient anxiety and postoperative maladaptive behavior score lowered (*p* = 0.015) No change in emergence delirium
ED	Kim et al. (2015) [51]	RCT	117	2–7	Video distraction and parental presence	Patient anxiety, parental anxiety, postoperative pain, emergence delirium, post-hospitalization behavior questionnaire	N/A	No change in emergence delirium
ED	Kain et al. (2007) [42]	RCT	408	2–10	ADVANCE intervention	Patient anxiety	Parental anxiety, emergence delirium, analgesic consumption, PACU length of stay	Lowered patient anxiety (*p* = 0.006) and emergence delirium after surgery (*p* = 0.038) in the recovery room (*p* = 0.016)

*ADVANCE intervention* anxiety reduction, distraction on the day of surgery, video modeling and education before the day of surgery, adding parents to the child’s surgical experience and promoting family-centered care, no excessive reassurance, coaching of parents by researchers to help them succeed, exposure/shaping of the child via induction mask practice, *BED* Bundle, bundle to eliminate delirium, *EA* emergence agitation, *ED* emergence delirium, *ICU* intensive care unit, *II/IH* ilioinguinal/iliohypogastric, *M* month, *Mypas* modified Yale Pre-operative Anxiety Scale, *N/A* not applicable, *OR* operating room, *PAED* Pediatric Anesthesia of Emergence Delirium, *PACU* post-anesthesia care unit, *PAD* pain, agitation, delirium, *PD* pediatric delirium, *PICU* pediatric intensive care unit, *PHBQ* Postoperative Maladaptive Behavior Questionnaire, *PPIA* parental presence during induction of anesthesia, *RCT* randomized controlled trial, *STAI-A* State-Trait Anxiety Inventory

### Classification and summary of non-pharmacological interventions

Table 2 presents the classification and summary of non-pharmacological delirium interventions. Non-pharmacological interventions were grouped into educational (*n* = 5), multicomponent (*n* = 6), and technology-assisted (*n* = 5).

**Table 2:** Non-pharmacological delirium interventions

Intervention type	First author (year)	Intervention summary
***Education***		
Delirium prevention toolkit & PICU journal	Silver and Traube (2019) [45]	The toolkit contained a pamphlet to educate the family about delirium To promote sleep, they included an eye mask to help eliminate light and headphones to reduce noise. The patients were encouraged to document their stay in a notebook (PICU Journal) to minimize PTSD
Health professional education	Rohlik et al. (2018) [30]	Education included information on general PD principles, delirium management strategies, and pCAM-ICU use
Pre-operative visit	Zhong et al. (2018) [31]	Children and parents visited the waiting area, operation room, and recovery room Pre-operative visit education encompassed the operation process, instruments, induction, and recovery process
Pre-operative preparation workshop	Hilly et al. (2015) [50]	Pre-operative education workshop explained the operation process, induction, and recovery process. Children became accustomed to the operating room using a scale model, Playmobil
Video-based PPIA preparation	Bailey et al. (2015) [29]	Parent education included what to expect in the OR, the role of parents, and the relationship between parental anxiety and children’s outcomes in the OR
***Multicomponent***		
PAD bundle	Cloedt et al. (2022) [43]	Assessments of pain and agitation were completed every 4 h Delirium screening was completed at 8–12 h using the CAPD Withdrawal assessment was performed every 12 h using the WAT-1
BED intervention	Rohlik et al. (2021) [44]	Day and night cycle was normalized, patients were oriented to their surroundings, and early mobility was promoted. The following were ensured in the study: provision of a familiar environment, avoidance of sensory over- or under-stimulation, and optimization of sleep BED paper checklists were created and placed in the patient’s room
BED bundle with noise pollution reduction	Kawai et al. (2019) [46]	Thirty-five sound sensors were installed in the patients’ bed spaces, hallway, and common area. The pediatric delirium bundle was implemented for over 28 days
ICU bundle	Simone et al. (2017) [48]	Delirium screening, prevention, and treatment: delirium screening using CAPD. Nurses and physicians were educated about CAPD. Monthly inter-professional case conferences increased delirium awareness. Sedation and early mobilization protocols were implemented
Video distraction and parental presence	Kim et al. (2015) [51]	Children watched cartoon videos with their parents throughout the whole anesthesia induction process
ADVANCE intervention	Kain et al. (2007) [42]	Anxiety reduction, distraction on the day of surgery, video modeling, and education before the day of surgery. Inclusion of parents in the child’s surgical experience, promotion of family-centered care, and no excessive reassurance. Exposure/shaping of the child via induction mask practice
***Technology-Assisted***		
Music intervention with application use	Garcia Guerra et al. (2021) [33]	The music group received classical music for 30 min three times a day through headphones. A music therapist selected pre-recorded short pieces of classical music
Unilateral right-side stimulation of HT7 acupuncture point	Nakamura et al. (2018) [47]	Unilateral right-side stimulation of the HT7 acupuncture point using a single twitch electrical stimulus was performed throughout the surgery
Mother’s voice	Byun et al. (2018) [32] Song et al. (2017) [34]	At the end of the operation, the recorded mother's voice was played through noise-cancelling headphones Maternal voice recordings were played repeatedly through headphones in the PACU
Ultrasound-guided II/IH nerve block	Ohashi et al. (2016) [49]	An anesthesiologist performed an ultrasound-guided nerve block Once the needle was placed between the internal oblique and transversus abdominus muscles, ropivacaine was injected

*PICU* pediatric intensive care unit, *PPIA* parental presence during anesthesia induction, *CAPD* Cornell Assessment of Pediatric Delirium, *OR* operating room, *PTSD* post-traumatic stress disorder, *PAD* pain, agitation, and delirium, *BED* Bundle to Eliminate Delirium, *WAT-1* Withdrawal Assessment Tool-1, *PACU* post-anesthesia care unit, *II/IH* ilioinguinal/iliohypogastric, *HT7* 7th acupoint of the heart meridian

#### Educational intervention

In the five studies on educational intervention, the intervention was conducted for pediatrics, their families, and healthcare professionals [29–31, 45, 50]. The children received educational intervention regarding the operation process, instruments, and induction and recovery processes during pre-operative visits [31]. Moreover, the children participated in a one-hour workshop that included group sessions explaining the pre-operative process [50]. The children could freely play with scale models and became accustomed to the surgical environment before surgery, and parents were allowed to participate in this entire session with their children [50].

Educational intervention demonstrated positive outcomes in reducing the incidence of emergence delirium, the dose of propofol administered during surgery (*p* < 0.05) [31], anxiety levels in children and parents (*p* < 0.01, mean difference [MD] -10 [-20; 0]), and postoperative maladaptive behavior (*p* < 0.008, MD = -2 [-3.3; -0.6]) [50]. Parents, instead of children, received educational intervention in two studies [29, 45]. As children have different developmental stages and recruiting them for intervention research in the hospital setting is challenging, researchers provided interventions to the parents on behalf of their children [29, 45]. Video-based delirium education was provided to parents using an iPad (Apple, Cupertino, CA) containing information regarding what to expect, the role of parents, and the relationship between parental anxiety and children’s outcomes in an operating room [29]. A delirium prevention toolkit containing a pamphlet on the importance of promoting a good night sleep was provided to the family [45]. Additionally, parents were encouraged to document the PICU journey in their diary to minimize post-traumatic stress disorder [45]. The educational intervention provided to parents did not reduce their child’s pre-operative anxiety and the occurrence of emergence delirium; however, parents in the intervention group reported higher self-efficacy in helping their child in the operating room (*p* = 0.03, Wilcoxon–Mann–Whitney odds ratio [95% confidence interval] = 1.69 [1.07–2.87]) [29].

Delirium education was also provided to healthcare professionals. The educational intervention encompassed the pediatric confusion assessment method for the ICU (pCAM-ICU), rationale for delirium assessment, documentation, and understanding of negative outcomes associated with delirium [30]. Early use of a quality improvement tool, comprehensive education, a monitoring system with feedback, and multidisciplinary team involvement led to an increase in the delirium screening rate from 51 to 71% [30].

#### Multicomponent interventions

Multicomponent interventions aim to alleviate delirium experiences in children through a sophisticated strategy involving multiple approaches administered in a bundle, encompassing delirium screening, prevention, and treatment in a combined manner. Kain et al. [42] created the ADVANCE bundle, focusing on anxiety reduction, distraction, video modeling, education, parental presence, less reassurance, and parent coaching. Cloedt et al. [43] developed the pain, agitation, delirium bundle, which actively assesses children’s pain and agitation every 4 h and delirium every 8–12 h using the Cornell assessment of pediatric delirium (CAPD). Rohlik et al. [44] developed the bundle to eliminate delirium (BED), encouraging normalization of day and night cycles, patient orientation to surroundings, and early mobility. Kawai et al. [46] combined the BED bundle with a noise pollution reduction intervention, utilizing sound sensors and implementing the BED bundle for over 28 days. Simone et al. [48] employed an ICU bundle comprising delirium screening, sedation protocols, and early mobilization protocols. A monthly interprofessional case conference increased awareness of delirium, with sedation and early mobilization protocols implemented using this bundle. These multicomponent non-pharmacological interventions increased delirium screening and detection rates, emphasizing the importance of early identification in pediatric delirium intervention and prevention. Moreover, the non-pharmacological interventions were simultaneously administered in a bundle approach. For instance, parental presence and video distraction were implemented concurrently [51]. However, the intervention did not lower the emergence delirium rate in children.

#### Technology-assisted interventions

Various technology-assisted non-pharmacological interventions have been used to lower the incidence of delirium in children, with varying effectiveness. In the study by Ohashi et al. [49], an anesthesiologist performed an ultrasound-guided nerve block. Nakamura et al. [47] intraoperatively applied unilateral right-side stimulation to the heart in seven acupuncture points using a single-twitch electrical stimulus to reduce emergence delirium in children. Byun et al. [32] and Song et al. [34] recorded mothers’ voices and played them through headphones, effectively lowering the children’s emergence agitation [34] and delirium scores (*p* = 0.006) [32]. However, another study playing classical music selected by a music therapist via headphones for 30 min three times a day did not report effectiveness in lowering pediatric delirium but showed positive outcomes in lowering sedation use in pediatric ICUs [33]. The choice of music also influenced the outcomes of the pediatric delirium intervention.

### Frequently measured outcome variables

Table 3 provides frequently measured outcome variables of pediatric and emergence delirium interventions. Pediatric delirium was measured using the CAPD [52] and pCAM-ICU in five studies [30, 33, 43, 44, 48]. In contrast, emergence delirium was measured using the Pediatric Anesthesia Emergence Delirium Scale (PAED) [53], Watcha scale [54], and Aono’s scale [55] in nine studies [29, 31, 32, 34, 42, 47, 49–51]. The most frequently measured outcome variable was pain, assessed using the Behavioral Observational Pain Scale [56], Face, Legs, Activity, Cry, and Consolability Scale [57], Comfort Behavioral Scale [58], and Visual Numeric Scale [59] in six studies [29, 32, 43, 47, 49, 51]. Patient anxiety was measured as an outcome variable using the modified Yale Pre-operative Anxiety Scale [42] in four studies [29, 42, 50, 51], and parental anxiety was measured using the State-Trait Anxiety Inventory [60] in four studies [29, 42, 50, 51]. PICU length of stay, agitation, analgesic consumption, and postoperative maladaptive behavior were also measured as outcome variables in different pediatric delirium research [31, 33, 34, 42, 43, 47, 50].

**Table 3:** Frequently measured outcome variables

Most measured outcome variables	Assessment tool	Use in studies
Emergence delirium	PAED, Watcha scale, Aono’s scale	Kain et al. (2007) [42], Bailey et al. (2015) [29], Hilly et al. (2015) [50], Kim et al. (2015) [51], Ohashi et al. (2016) [49], Song et al. (2017) [34], Byun et al. (2018) [32], Nakamura et al. (2018) [47], Zhong et al. (2018) [31]
Pediatric delirium	CAPD, p CAM ICU	Simone et al. (2017) [48], Rohlik et al. (2018) [30], Cloedt et al. (2022) [43] Garcia Guerra et al. (2021) [33], Rohlik et al. (2021) [44]
Patient anxiety	mYPAS	Kain et al. (2007) [42], Bailey et al. (2015) [29], Hilly et al. (2015) [50], Kim et al. (2015) [51]
Parent anxiety	STAI-A	Kain et al. (2007) [42], Bailey et al. (2015) [29], Hilly et al. (2015) [50], Kim et al. (2015) [51]
Pain	BOPS, FLACC, COMFORT-B, VNS	Bailey et al. (2015) [29], Kim et al. (2015) [51], Ohashi et al. (2016) [49], Byun et al. (2018) [32], Nakamura et al. (2018) [47], Cloedt et al. (2022) [43]
Postoperative maladaptive behavior	PHBQ	Hilly et al. (2015) [50], Kim et al. (2015) [51]
Agitation	RASS, WAT-1, Watcha scale	Song et al. (2017) [34], Zhong et al. (2018) [31], Cloedt et al. (2022) [43]
PACU/PICU length of stay	N/A	Kain et al. (2007) [42], Hilly et al. (2015) [50], Song et al. (2017) [34], Nakamura et al. (2018) [47]
Analgesics consumption	N/A	Kain et al. (2007) [42], Hilly et al. (2015) [50], Cloedt et al. (2022) [43], Garcia Guerra et al. (2021) [33]

*BOPS* Behavioral Observational Pain Scale, *CAPD* Cornell Assessment of Pediatric Delirium, *COMFORT-B* Comfort Behavioral Scale, *FLACC* Face, Legs, Activity, Cry and Consolability, *mYPAS* Modified Yale Pre-operative Anxiety Scale, *N/A* not applicable, *PHBQ* Postoperative Maladaptive Behavior Questionnaire, *PAED* Pediatric Anesthesia of Emergence Delirium, *P CAM ICU* Pediatric Confusion Assessment Method for Intensive Care Unit Patients, *RASS* Richmond Agitation Sedation Scale, *STAI-A* State-Trait Anxiety Inventory, *VNS* Visual Numeric Scale, *WAT-1* Withdrawal Assessment Tool-1

### Quality appraisal

**Fig. 2:**
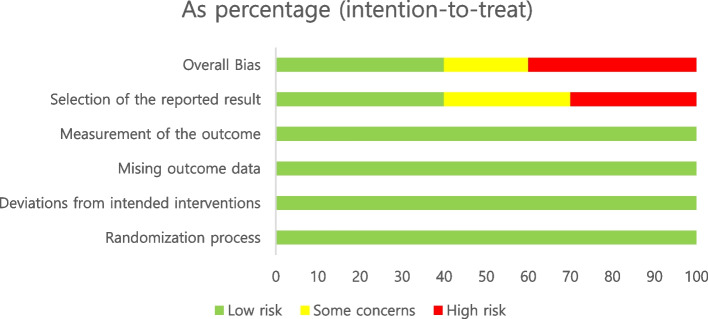
Risk of bias for included studies

Two authors independently conducted quality appraisals of all included studies. According to the Joanna Briggs Institute (JBI) critical appraisal checklist for randomized control trials (Supplementary Table 2), five studies were reported to have a moderate risk of bias [29, 30, 33, 42, 51], while five studies were reported to have a low risk of bias [31, 32, 34, 47, 49]. Using the JBI critical appraisal checklist for cohort studies, one cohort study was reported to have a low risk of bias [43], and the other had a moderate risk of bias [50] (Supplementary Table 3). Four studies [44–46, 48] were evaluated using the Quality Improvement Minimum Quality Criteria (QI-MQCS) (Supplementary Table 4). Overall, the risk of bias in the included studies was rated as “some concern” and “high risk” (Fig. 2). Due to the diversity in the types of studies included, various quality improvement tools were used.

## Discussion

Non-pharmacological interventions, including educational, multicomponent, and technology-assisted approaches, were implemented to alleviate pediatric delirium and emergence delirium in hospitalized children. Even though the outcomes varied, these interventions suggest potential for further development in future research. Multicomponent interventions, particularly the bundle approach, demonstrated effectiveness in delirium detection, highlighting the importance of early prevention and intervention. Educational interventions yielded more favorable outcomes when directly provided to children and healthcare professionals than to parents. Contrarily, technology-assisted interventions involving invasive procedures showed limited effectiveness, whereas recording the mother’s voice was helpful.

Recent multicomponent delirium interventions have demonstrated effectiveness in adult and pediatric patients across various hospital settings [61–66]. This aligns with the findings of this systematic review, indicating that the bundle approach is associated with increased delirium screening and detection rates. Thus, compared with a single-intervention approach in children [33, 47, 49], multicomponent approaches allow for simultaneous delirium screening, detection, and treatment [48].

The outcomes of the educational interventions varied according to the target group. Direct educational interventions for children yielded positive results, reducing the incidence of emergence delirium, propofol administration during surgery (*p* < 0.05) [31], children’s and parent’s anxiety levels, and postoperative maladaptive behavior (*p* = 0.015) [50]. In essence, hands-on educational experiences at “eye level,” considering the child’s perspective, proved to be a crucial factor for the success of educational interventions [31, 50]. Future studies should consider developing age-specific strategies, as indicated by key findings in positive outcomes [30, 48, 67].

Educational interventions provided to healthcare professionals were equally successful. This success is attributed to healthcare professionals being the primary caregivers in the PICU and operating room, where parents may not be present with their children. Improved knowledge, self-confidence, and attitude toward delirium assessment and management among healthcare professionals contributed to shorter times for delirium diagnosis and ensured early intervention [44, 68]. Additionally, a multidisciplinary team approach in hospitals reduced barriers to delirium screening among children [30]. This positive cycle resulted in improved outcomes in managing delirium in children.

Conversely, educational interventions provided to parents were ineffective. This ineffectiveness could be attributed to the medical environment in which delirium occurred. The current medical environment frequently shows limitations in incorporating family centered care and parental partnerships in pediatric healthcare [69], which is a key component of delirium intervention in children [62]. Children are separated from their parents during their stay in the PICU or surgery. Delirium education provided to parents may yield partial results, as they do not have enough time or opportunity to positively influence their children. These thoughts would explain the effects of recorded maternal voices on reducing emergency delirium after surgery [70]. Future delirium interventions should carefully consider these aspects and enhance the parent–child relationship.

Technology-assisted interventions have also been explored in hospitalized children. Recordings of mothers’ voices played through headphones were an effective and non-invasive intervention approach for children [34]. Conversely, stimulating acupuncture points and performing nerve blocks are invasive procedures for children [47, 49], showing limited evidence in lowering pediatric or emergence delirium. This raises questions about whether a medical intervention, particularly an invasive approach, is the most suitable method for addressing the complex causes and symptoms of delirium in children. In future research, careful consideration should be given to interventions that minimize harm while maximizing positive outcomes for children.

A difference was observed in the method of research between pediatric and emergence delirium. Non-pharmacological interventions for emergence delirium in children were mostly used in RCTs, whereas non-pharmacological interventions for pediatric delirium were used in quality improvement projects and one cohort study [43]. Quality improvement projects do not offer the highest quality of research evidence; however, we verified the included quality improvement interventions using the quality improvement minimum quality criteria set [71] in this systematic review. These quality improvement reports are sufficient to serve as a basis for superior forms of pediatric delirium research in the future. We anticipate that the level of non-pharmacological intervention research on pediatric delirium will increase based on these quality improvement projects. This review highlights the need for RCTs in future pediatric and emergence delirium research.

In particular, in the research setting of pediatric delirium the main challenges are the lack of standardized terminology, dedicated assessment tools, and outcome measures. The major impediment is the current inability of assessment tools to differentiate between pediatric delirium subtypes [8]. Commonly used tools, such as CAPD and PAED, have limitations, including age-specific specificity and sensitivity issues, making the evaluation challenging for delirium subtypes [52, 72, 73]. Furthermore, the global lack of awareness regarding the substantial difference between hyperactive and hypoactive delirium in children poses another impediment to delirium treatment in this population [74]. The hypoactive subtype of pediatric delirium is clinically distinct, associated with worse long-term outcomes, and is unresponsive to drug treatment [75]. This limitation is critical because neglecting subtype differences may lead to incorrect uniform treatment approaches, including pharmacological interventions [8]. Antipsychotic medications are frequently applied universally despite discussions highlighting their ineffectiveness, particularly in hypoactive delirium [76]. Recent critical care research has explored diverse delirium biomarkers to address these issues [77]. Delivering effective tailored interventions for future pediatric delirium cases requires further research delving into the symptom science of pediatric delirium, facilitating the need for accurate subtyping of delirium in children.

Delirium in children is characterized by a cluster of symptoms with multifactorial etiologies [61], which pose challenges in distinguishing it from fear, anxiety, and agitation [6]. The diverse developmental ages and cognitive abilities of children in the PICU further complicate delirium assessment [78]. Faced with these challenges, some researchers have prioritized fear, anxiety, and agitation as primary outcomes and treated pediatric or emergency delirium as a secondary outcome [29, 33, 42, 50]. However, this approach has a critical limitation in that it may lead to inaccurate reporting because of the potential overlap of primary and secondary outcomes. Future research should focus on identifying appropriate outcome variables that are distinct from other psychological symptoms to precisely assess the effectiveness of interventions in non-pharmacological studies.

### Limitations

This study has some limitations. We aimed to conduct an integrated analysis; however, statistical analysis of the resulting data was not possible. Furthermore, the heterogeneity of the included studies prevented us from conducting a meta-analysis of the included studies. Therefore, we performed a narrative synthesis of the included studies. Additionally, we only included articles written in English, which could have limited our study findings. As shown in Fig. 2, this systematic review included research with a high risk of bias, which was nevertheless incorporated into the narrative synthesis due to the limited research on delirium interventions in children.

## Conclusions

Delirium is a significant complication observed in many hospitalized children. Various non-pharmacological interventions, including educational, multicomponent, and technology-assisted approaches, are being explored to mitigate pediatric and emergence delirium in hospitalized children. Although direct comparisons of intervention effectiveness may be challenging owing to different outcome variables, our study highlights the efficacy and limitations of non-pharmacological interventions in pediatric delirium. Additional research is crucial to further enhance our understanding. We advocate for studies using standardized delirium and outcome measurement tools to enable quantitative comparisons, contributing to the advancement of knowledge and the enhancement of care quality for children experiencing delirium.

### Supplementary Information

**Additional file 1: Supplementary Table 1.** Literature search strategy.

**Additional file 2: Supplementary table 2.** Joanna Briggs Institute (JBI) critical appraisal checklist for randomized control trials.

**Additional file 3: Supplementary table 3.** Joanna Briggs Institute (JBI) critical appraisal checklist for cohort studies.

**Additional file 4: Supplementary table 4.** Quality improvement minimum quality criteria (QI-MQCS).

## Data Availability

The datasets used during the current study are available from the corresponding author on reasonable request.

## Abbreviations

ADVANCE intervention: Anxiety reduction, distraction on the day of surgery, video modeling and education before the day of surgery, adding parents to the child’s surgical experience and promoting family-centered care, no excessive reassurance, coaching of parents by researchers to help them succeed, exposure/shaping of the child via induction mask practice
BED: Bundle to Eliminate Delirium
CAPD: Cornell Assessment of Pediatric Delirium
CINAHL: Cumulative Index to Nursing and Allied Health Literature
EMBASE: Excerpta Medica database
ICU: Intensive care unit
MD: Mean difference
PAED: Pediatric Anesthesia of Emergence Delirium
PACU: Post-anesthesia care unit
PICU: Pediatric intensive care unit
pCAM-ICU: Pediatric Confusion Assessment Method for Intensive Care Unit Patients
RCT: Randomized controlled trial
