# Supplementary material for: Non-pharmacological interventions for delirium in the pediatric population: a systematic review with narrative synthesis
Source: BMC Pediatr. 2024 Feb 12;24:108. doi: 10.1186/s12887-024-04595-4 (PMC10863154; doi:10.1186/s12887-024-04595-4)
Supplement: Supplementary file 1 — Additional file 1: Supplementary Table 1. Literature search strategy. [file 12887_2024_4595_MOESM1_ESM.docx]

**Non-pharmacological Interventions for Delirium in the Pediatric Population: A Systematic Review with Narrative Synthesis: Kyua KIM, MSN, RNa, Ju Hee JEONG, MSN, RNb, Eun Kyoung CHOI, PhD, RN, CPNPc**

**College of Nursing & Mo-Im Kim Nursing Research Institute, Yonsei University, 50-1 Yonsei-ro, Seodaemun-gu, Seoul 03722, South Korea. ekchoi@yuhs.ac**

**Supplementary Table 1. Literature search strategy**

| Database  (No time limit) | Search terms | N |
| --- | --- | --- |
| PubMed | (("child" OR "children" OR "pediatric*" OR "newborn" OR "infant" OR "child" (mesh) OR "adolescent" (mesh) OR "newborn" (mesh) OR "infant" (mesh)) AND (("delirium" OR "delirium" (MESH)) AND (intervention OR management OR "methods"[MeSH Terms] OR organization AND administration[MeSH Terms] OR disease management[MeSH Terms])) | 406 |
| Web of Science | (("child" OR "children" OR "pediatric*" OR "adolescent" OR "newborn" OR "Neonate" OR "infant") AND ("delirium") AND ("intervention" OR "management")) | 468 |
| CINAHL | (("child*" OR "children" OR "pediatric*" OR "adolescent*" OR "newborn" OR "neonate" OR "infant" OR "MH infant" OR "MH adolescence" OR "MH child*") AND (“delirium” OR "MH delirium") AND (“intervention” OR “management” OR “MH intervention” OR “MH management”)) | 151 |
| EMBASE | (("child*" OR "children" OR "pediatric*" OR "adolescent" OR "newborn" OR "neonate" OR "infant" OR child/exp OR newborn/exp OR adolescent/exp) AND (“delirium” OR delirium/exp) AND (intervention OR management OR 'intervention'/exp OR 'management'/exp)) | 854 |
|  |  |  |
