# Supplementary material for: Non-pharmacological interventions for delirium in the pediatric population: a systematic review with narrative synthesis
Source: BMC Pediatr. 2024 Feb 12;24:108. doi: 10.1186/s12887-024-04595-4 (PMC10863154; doi:10.1186/s12887-024-04595-4)
Supplement: Supplementary file 2 — Additional file 2: Supplementary table 2. Joanna Briggs Institute (JBI) critical appraisal checklist for randomized control trials. [file 12887_2024_4595_MOESM2_ESM.docx]

**Non-pharmacological Interventions for Delirium in the Pediatric Population: A Systematic Review with Narrative Synthesis**

**Kyua KIM, MSN, RNa, Ju Hee JEONG, MSN, RNb, Eun Kyoung CHOI, PhD, RN, CPNPc**

**College of Nursing & Mo-Im Kim Nursing Research Institute, Yonsei University, 50-1 Yonsei-ro, Seodaemun-gu, Seoul 03722, South Korea. ekchoi@yuhs.ac**

**Supplementary table 2. Joanna Briggs Institute (JBI) critical appraisal checklist for randomized control trials**

| Study | Q1 | Q2 | Q3 | Q4 | Q5 | Q6 | Q7 | Q8 | Q9 | Q10 | Q11 | Q12 | Q13 | Risk bias |
| --- | --- | --- | --- | --- | --- | --- | --- | --- | --- | --- | --- | --- | --- | --- |
| Garcia (2021) | Y | Y | N | Y | N | N | Y | Y | Y | Y | Y | Y | Y | Moderate |
| Byun (2018) | Y | Y | Y | Y | Y | Y | Y | Y | Y | Y | Y | Y | Y | Low |
| Nakamura (2018) | Y | Y | Y | Y | Y | Y | Y | Y | Y | Y | Y | Y | Y | Low |
| Rohlik (2018) | UC | UC | UC | Y | Y | Y | Y | Y | Y | Y | Y | Y | Y | Moderate |
| Zhong (2018) | Y | Y | Y | UC | UC | Y | Y | Y | Y | Y | Y | Y | Y | Low |
| Song (2017) | Y | Y | Y | N | N | N | Y | Y | Y | Y | Y | Y | Y | Low |
| Ohashi (2016) | Y | Y | Y | Y | Y | Y | Y | Y | Y | Y | Y | Y | Y | Low |
| Bailey (2015) | Y | Y | Y | N | N | UC | Y | N | Y | Y | Y | Y | Y | Moderate |
| Kim (2015) | Y | Y | N | Y | UC | Y | Y | UC | N | Y | Y | Y | Y | Moderate |
| Kain (2007) | Y | Y | Y | UC | N | UC | N | UC | Y | Y | Y | Y | Y | Moderate |

Key: Y = yes; N = no; UC = unclear

Q1. Was true randomization used for assignment of participants to treatment groups?

Q2. Was allocation to treatment groups concealed?

Q3. Were treatment groups similar at the baseline?

Q4. Were participants blind to treatment assignment?

Q5. Were those delivering treatment blind to treatment assignment?

Q6. Were outcomes assessors blind to treatment assignment?

Q7. Were treatment groups treated identically other than the intervention of interest?

Q8. Was follow up complete and if not, were differences between groups in terms of their follow up adequately described and analyzed?

Q9. Were participants analyzed in the groups to which they were randomized?

Q10.Were outcomes measured in the same way for treatment groups?

Q11. Were outcomes measured in a reliable way?

Q12. Was appropriate statistical analysis used?

Q13. Was the trial design appropriate, and any deviations from the standard RCT design (individual randomization, parallel groups) accounted for in the conduct and analysis of the trial?
