# Supplementary material for: Non-pharmacological interventions for delirium in the pediatric population: a systematic review with narrative synthesis
Source: BMC Pediatr. 2024 Feb 12;24:108. doi: 10.1186/s12887-024-04595-4 (PMC10863154; doi:10.1186/s12887-024-04595-4)
Supplement: Supplementary file 3 — Additional file 3: Supplementary table 3. Joanna Briggs Institute (JBI) critical appraisal checklist for cohort studies. [file 12887_2024_4595_MOESM3_ESM.docx]

**Non-pharmacological Interventions for Delirium in the Pediatric Population: A Systematic Review with Narrative Synthesis**

**Kyua KIM, MSN, RNa, Ju Hee JEONG, MSN, RNb, Eun Kyoung CHOI, PhD, RN, CPNPc**

**College of Nursing & Mo-Im Kim Nursing Research Institute, Yonsei University, 50-1 Yonsei-ro, Seodaemun-gu, Seoul 03722, South Korea. ekchoi@yuhs.ac**

**Supplementary table 3. Joanna Briggs Institute (JBI) critical appraisal checklist for cohort studies**

| Study | Q1 | Q2 | Q3 | Q4 | Q5 | Q6 | Q7 | Q8 | Q9 | Q10 | Q11 | Q12 | Risk bias |
| --- | --- | --- | --- | --- | --- | --- | --- | --- | --- | --- | --- | --- | --- |
| Cloedt (2022) | Y | Y | Y | Y | Y | UC | Y | Y | Y | Y | Y | Y | Low |
| Hilly (2015) | Y | N | N | Y | Y | Y | UC | UC | Y | Y | N | Y | Moderate |

Key: Y = yes; N = no; UC = unclear

Q1. Were the two groups similar and recruited from the same population?

Q2. Were the exposures measured similarly to assign people?

Q3. Were the exposures measured similarly to both exposed and unexposed groups?

Q4. Was the exposure measured in a valid and reliable way?

Q5. Were confounding factors identified?

Q6. Were strategies to deal with confounding factors stated?

Q7. Were the groups/participants free of the outcome at the start of the study (or at the moment of exposure)?

Q8. Were the outcomes measured in a valid and reliable way?

Q9. Was the follow up time reported and sufficient to be long enough for outcomes to occur?

Q10. Was follow up complete, and if not, were the reasons to loss to follow up described and explored?

Q11. Were strategies to address incomplete follow up utilized?

Q12. Was appropriate statistical analysis used?
