# Supplementary material for: Non-pharmacological interventions for delirium in the pediatric population: a systematic review with narrative synthesis
Source: BMC Pediatr. 2024 Feb 12;24:108. doi: 10.1186/s12887-024-04595-4 (PMC10863154; doi:10.1186/s12887-024-04595-4)
Supplement: Supplementary file 4 — Additional file 4: Supplementary table 4. Quality improvement minimum quality criteria (QI-MQCS). [file 12887_2024_4595_MOESM4_ESM.docx]

**Non-pharmacological Interventions for Delirium in the Pediatric Population: A Systematic Review with Narrative Synthesis**

**Kyua KIM, MSN, RNa, Ju Hee JEONG, MSN, RNb, Eun Kyoung CHOI, PhD, RN, CPNPc**

**College of Nursing & Mo-Im Kim Nursing Research Institute, Yonsei University, 50-1 Yonsei-ro, Seodaemun-gu, Seoul 03722, South Korea. ekchoi@yuhs.ac**

**Supplementary table 4. Quality improvement minimum quality criteria (QI-MQCS)**

| Study | D1 | D2 | D3 | D4 | D5 | D6 | D7 | D8 | D9 | D10 | D11 | D12 | D13 | D14 | D15 | D16 |
| --- | --- | --- | --- | --- | --- | --- | --- | --- | --- | --- | --- | --- | --- | --- | --- | --- |
| Rohlik (2021) | ✔ | ✔ | ✔ | ✔ | ✔ | ✔ |  | ✔ | ✔ | ✔ | ✔ | ✔ | ✔ | ✔ | ✔ | ✔ |
| Silver (2019) | ✔ | ✔ | ✔ | ✔ | ✔ | ✔ |  |  |  | ✔ |  | ✔ | ✔ | ✔ | ✔ | ✔ |
| Kawai (2019) | ✔ | ✔ | ✔ | ✔ | ✔ | ✔ | ✔ | ✔ | ✔ | ✔ |  | ✔ | ✔ |  | ✔ | ✔ |
| Simone (2017) | ✔ | ✔ | ✔ | ✔ | ✔ | ✔ |  | ✔ | ✔ | ✔ | ✔ | ✔ | ✔ | ✔ | ✔ | ✔ |

Domain

D1. Organizational Motivation, D2. Intervention Rationale, D3. Intervention Description, D4. Organizational Characteristics,

D5. Implementation, D6. Study Design, D7. Comparator, D8. Data Source, D9. Timing, D10. Adherence/ Reach, D11. Health Outcome

D12. Organizational Readiness, D13. Penetration/Reach, D14. Sustainability, D15. Spread, D16. Limitation
